# Supplementary material for: Polyketide synthase gene clusters in the ericoid mycorrhizal fungus Oidiodendron maius: genome-wide prediction, expression profiling, and functional characterization of knockout mutants
Source: Front Plant Sci. 2026 Sep 16;17:1924430. doi: 10.3389/fpls.2026.1924430 (PMC13623903; doi:10.3389/fpls.2026.1924430)
Supplement: Supplementary file 1 [file SupplementaryFile1.zip › Supplementary Materials/Supplementary Folder 1.zip!/Oidma1_AssemblyScaffolds_Repeatmasked/knownclusterblast/cluster58/jgi.p_Oidma1_46577_mibig_hits.html]

| MIBiG Protein | Description | MIBiG Cluster | MiBiG Product | % ID | % Coverage | BLAST Score | E-value |
| --- | --- | --- | --- | --- | --- | --- | --- |
| EPE34350.1 | Clavaminate\_synthase-like\_protein | BGC0001035 | Pneumocandin\_biosynthetic\_gene\_cluster | 43 | 106.07028754 | 291 | 1.3e-78 |
| AGA37270.1 | 2OG-Fe(II)-oxygenase | BGC0000819 | Paraherquamide\_biosynthetic\_gene\_cluster | 40 | 105.111821086 | 269 | 6.9e-72 |
| AFT91382.1 | EcdK | BGC0000338 | Echinocandin\_B\_biosynthetic\_gene\_cluster | 41 | 106.07028754 | 264 | 1.7e-70 |
| AMM63173.1 | AniK | BGC0001371 | Echinocandin\_B\_biosynthetic\_gene\_cluster | 39 | 106.07028754 | 258 | 9.3e-69 |
| AMM63174.1 | AniF | BGC0001371 | Echinocandin\_B\_biosynthetic\_gene\_cluster | 33 | 105.431309904 | 200 | 3e-51 |
| AFT91391.1 | HtyE | BGC0000338\_c2 | Echinocandin\_B\_biosynthetic\_gene\_cluster | 34 | 105.431309904 | 194 | 1.7e-49 |
